# Anthocyanins in Strawberry Polyphenolic Extract Enhance the Beneficial Effects of Diets with Fructooligosaccharides in the Rat Cecal Environment

**DOI:** 10.1371/journal.pone.0149081

**Published:** 2016-02-16

**Authors:** Bartosz Fotschki, Jerzy Juśkiewicz, Adam Jurgoński, Krzysztof Kołodziejczyk, Joanna Milala, Monika Kosmala, Zenon Zduńczyk

**Affiliations:** 1 Division of Food Science, Institute of Animal Reproduction and Food Research, Polish Academy of Sciences, Olsztyn, Poland; 2 Institute of Food Technology and Analysis, Łódź University of Technology, Łódź, Poland; Columbia University, UNITED STATES

## Abstract

The administration of fructooligosaccharides (FOS) beneficially modulates gastrointestinal functions and may enhance the metabolism of polyphenols. However, different polyphenolic components in the diet may have different influences on the activities of the digestive enzymes and microbiota in the gastrointestinal tract. Therefore, a 4-week study of forty-eight male Wistar rats was conducted to investigate the physiological response of the rat cecal environment to diets without and with FOS that contained two different strawberry polyphenolic extracts, specifically EP (polyphenolic profile 60, 35, 5, and 0% ellagitannins, proanthocyanidins, flavonols, anthocyanins, respectively) and EPA (polyphenolic profile: 50, 35, 6, and 9%, respectively). When combined with FOS, both extracts beneficially enhanced the acidification of the cecal digesta (P≤0.05 vs the groups without extracts), but the dietary combination of EPA and FOS elicited the greatest reduction in putrefactive short-chain fatty acid production and the lowest fecal β-glucuronidase activity in the cecum (P≤0.05 vs group EP). Moreover, the addition of dietary FOS elevated the metabolism of the examined strawberry extracts in the cecum and thereby increased the concentrations of the metabolites in the cecal digesta and urine (P≤0.05 vs the group with cellulose). Overall, both strawberry extracts modulated the effects of FOS in the gastrointestinal tract; however, the combination with EPA extract that contained anthocyanins exhibited greater beneficial effects in the lower gut environment than the EP extract.

## Introduction

Fructooligosaccharides (FOS) are a specific group of linear fructans that occur in many plants. These compounds are a constituent of dietary fiber, are broken down by specific bacteria in the hindgut and are categorized as substances with prebiotic properties [1]. The administration of FOS beneficially modulates gastrointestinal functions by, e.g., increasing the production of short-chain fatty acids (SCFAs), primarily butyrate, which is an energy substrate for colonocytes [1]. Moreover, FOS decreases the activity of bacterial β-glucuronidase, which supports the undesirable transformation of xenobiotics into toxic substances [2]. Furthermore, the consumption of dietary FOS may enhance the metabolism of polyphenols [3, 4]. Metabolites, such as those from ellagitannins (ETs), may have favorable effects on the levels and proportions of cholesterol fractions, blood lipid levels, and vascular inflammation [5, 6]. In contrast, a previous study demonstrated that a diet enriched with ETs may thwart some beneficial effects of FOS in the gastrointestinal tract and lipid profile in the serum [4]. Currently, little information about the interaction between polyphenols and FOS in the gastrointestinal tract is available.

Strawberries are an interesting source of polyphenols, particularly ETs, anthocyanins (ACs) and proanthocyanidins (PACs) [7]. ETs exhibit many positive effects on human health that are primarily due to their antioxidant, anti-neurodegenerative, and anti-inflammatory effects [5, 8]. Furthermore, there is considerable current interest in the possible health effects of ACs and PACs in humans due to their potential antioxidant effects and their reported positive effects on blood vessels [9]. Moreover, these polyphenols may play important roles in regulating digesting enzymes and the activity of the microbiota that live in the lower gut [10]. Some studies have reported that the majority of dietary ACs and ETs are not absorbed in the upper parts of the gastrointestinal tract; thus, they reach the colon and are metabolized by intestinal microbiota, which results in the generation of new compounds that may be absorbed and can modulate the activity of the microbiota [4, 10]. Moreover, PACs have been observed to inhibit the activities of digestive enzymes and may have important local functions in the gut [11, 12]. Our previous studies on rats revealed that polyphenol-rich extracts modulate the activities of the gastrointestinal endogenous enzymes and the production of SCFAs [4, 13].

Different polyphenolic components in the diet may have different influences on the activities of digestive enzymes and the microbiota in the gastrointestinal tract [10, 14]. Therefore, the aim of this study was to identify the combination of dietary FOS and two strawberry extracts containing different concentrations of ETs, PACs and ACs that most effectively elevated the beneficial effects in the lower gut environment. Moreover, the effects of FOS on the metabolism of strawberry polyphenols in the gastrointestinal tract were evaluated.

## Materials and Methods

### Preparation of the EP strawberry extract

Strawberry press cakes (750 kg) were collected from a strawberry juice production line of the Alpex Company (Łęczeszyce, Poland) and dried at 70±2°C. After drying to 400 kg, the press cakes were separated via the use of appropriate screens into a seed fraction (diameter 0.5–1 mm) and a seedless fraction (diameter 1–3 mm).

The raw polyphenol extracts were obtained from the seedless fraction via alcohol and acetone extraction. 6 kg of seedless fraction and 20 l of 65% ethanol in water were put in stainless steel 30 l volume extractor. The mixture was left for 48 hours at 20–25°C, next was separated on laboratory press resulting in 14.7 l of ethanol extract and 10.2 kg of wet pomace. The solvent was recovered by distillation, which gave 6 l of residue. The residue was rich in polyphenols and contained 15% of ethanol. 10.2 kg of wet pomace after first extraction was mixed with 15 l of 65% acetone in water and put in the extractor at 20°C for 24 hours. After the second extraction 15 l of aceton-ethanol extract was separated from 10 kg of pomace (wet weight) on laboratory press. Resulted 10 kg of pomace was mixed with 10 l of water and pressed after 1 hour to result in 11 kg of wet pomace and 8 l of acetone-ethanol-water extract. Both acetone extract were joined (15 l and 8 l) and rectified with acetone and ethanol recovery, and resulted in 6 l of residue containing c.a. 15% of ethanol. The residue was joined with the residue from first extraction. 12 l of extract containing 600 g of dry matter were filtered on cellulose filter next subjected to purification. Joined extracts that were next purified on an adsorbent Amberlite XAD in 20 l column with 15 l of the adsorbtion bed. The process consisted of sorbent conditioning as recommended by bed manufacturer, adsorption of the polyphenols in the column bed with flow speed 1 BV/h (BV–bed volume), washing the low molecular weight saccharides and ions off of the bed with the same flow rate by the use of 2 BV of 8% of ethanol in water, and successive desorption of the polyphenols fractions with opposite flow direction, flow rate 0.2 BV/h and increasing ethanol concentration, i.e. 30%–1 BV, and 55%—until the desorption is completed. During the desorption 0.1 BV fractions were collected and analyzed for polyphenols. The fractions of similar compositions were joined, concentrated and freeze-dried. The following fractions that were distinguished by the proportions of primary polyphenol groups: the ETs, the PACs, the ACs, and the flavan-3-ols. The resulting lyophilized products were placed in PET containers and stored at -4°C in the absence of light. The nutritional components and polyphenols of the individual products were determined.

### Preparation of the EPA strawberry extract

This strawberry polyphenolic extract was enriched in ACs and was obtained from 50 kg of concentrated (62°Bx) fresh, commercial product strawberry juice (batch 12 06 13). Strawberry juice is significantly richer source of ACs, particularly pelargonidin glycosides, than strawberry press cake. The concentration of pelargonidin-3-glucoside in the juice used for the preparation was 1.1 g/kg of the dry weight of the concentrated strawberry juice. The juice was diluted to 25°Bx and used (two portions of 55 l) for absorption on a column in two consecutive batches. The absorption was performed as described above in the procedure for the separation of the polyphenols from press cake extracts.

### Basic chemical composition

The dry matter, ash, crude protein, crude fat, and total dietary fiber (TDF) were determined according to the official Association of Official Analytical Chemists (AOAC) methods (2005) 920.151, 940.26, 920.152, 930.09, and 985.29, respectively [15]. The carbohydrate contents were determined using the following formula: carbohydrate = total solids–(protein + fat + ash).

### The polyphenol contents

The concentrations of the ETs, ellagic acid, ACs and flavonols in the extracts were determined following their dilution in methanol (1 mg/mL) using a HPLC (Knauer Smartline system with a photodiode array detector, Berlin, Germany) coupled with a Gemini C18 column (110 Å, 250×4.60 mm; 5 μm, Phenomenex, Torrance, USA). Phase A was 0.05% phosphoric acid in water, phase B was 0.05% phosphoric acid in 80% acetonitrile, the flow rate was 1.25 mL/min, the sample volume was 20 μL, and the temperature was 35°C. The following gradient was applied: stabilization for 5 min with 4% phase B, 4–15% B for 5–12.5 min, 15–40% B for 12.5–42.5 min, 40–50% B for 42.5–51.8 min, 50–55% B for 51.8–53.4 min, and 4% B for 53.4–55 min. The following standards were used for the identification of the polyphenols: ellagic acid, flavonols (quercetin-3-O-glucoside, kaempferol-3-O-glucoside, quercetin, kaempferol, and tiliroside), pelargonidin-3-O-glucoside (all from Extrasynthese, Genay, France), *p*-coumaric acid (Sigma-Aldrich), and samples of the ETs specifically hexahydroxydiphenoyl-d-glucose and agrimoniin, which were by semi-preparative HPLC as described by Sójka et al. (2013) [7]. The absorbances were measured at 280 nm (for *p*-coumaric acid, tiliroside, bishydroxydiphenoyl-d-glucose and agrimoniin), 360 nm (for ellagic acid, quercetin, kaempferol and kaempferol glycosides) and 520 nm (for the ACs).

The concentration of PACs in the extracts was determined by the HPLC method following PAC breakdown in an acidic environment with an excess of phloroglucinol according to the methods of Kennedy and Jones (2001) [16]. The obtained breakdown products were separated using a Knauer Smartline chromatograph (Berlin, Germany) equipped with an UV–Vis detector (PDA 280, Knauer, Berlin, Germany) and a fluorescence detector (Shimadzu RF-10Axl, Kyoto, Japan) and coupled with a Gemini C18 column (110 Å, 250×4.60 mm; 5 μm, Phenomenex, Torrance, USA). The separation conditions have previously been described by Kosmala et al. (2015) [17]. The identification was performed at 280 nm using a UV–Vis detector and the following standards: (–)-epicatechin, (+)-catechin, (–)-epigallocatechin, and their respective phloroglucinol adducts. Quantification was conducted based on the peak areas that were registered by a fluorescence detector (excitation wavelength: 278 nm; emission wavelength: 360 nm). The standard curves of (–)-epicatechin and (+)-catechin and the (–)-epicatechin-phloroglucinol adduct were used to quantify the breakdown products of the terminal units and extender units, respectively. The chemical and polyphenolic compositions of the strawberry extracts are presented in Table 1.

**Table 1:** Basic chemical and polyphenolic compositions of the strawberry extracts.

	Extract EP	Extract EPA
	%	
Dry matter	93.3	99.1
Ash	0.36	0.05
Fat	0.0	0.0
Crude protein	2.73	5.23
Carbohydrates^1^	18.3	65.3
including SDF	0.0	0.0
Polyphenols (HPLC-DAD)	71.8	28.6
Ellagic acid	0.7	0.3
Ellagitannins	43.2	13.7
monomers	23.5	7.4
dimers	19.7	6.4
Proanthocyanidins	24.8	10.1
Flavonols	3.2	1.9
Anthocyanins	0.0	2.6

^1^Low-molecular carbohydrates and structural components of plant cell walls.

### Quantification of ellagic acid and the ET metabolites

The ellagic acid concentration was determined in the cecal digesta following the hydrolysis of the digesta with trifluoroacetic acid. The digesta (0.2 mg) was mixed with 70% glycerol (0.5 mL) and 75 μL of trifluoroacetic acid and incubated at 95°C for 18 h. Subsequently, the sample was cooled and extracted 3 times using 1.5 mL of methanol in an ultrasonic bath. After each extraction, the sample was centrifuged (3 min, 10,000 *g*), and the supernatant was collected in a volumetric flask that was then filled with methanol. The ellagic acid content was then determined using HPLC (a Knauer Smartline system with a photodiode array detector; Berlin, Germany) coupled with a Gemini C18 column (110 Å, 250×4.60 mm; 5 μm, Phenomenex, Torrance, USA). Phase A consisted of 0.05% phosphoric acid in water, phase B consisted of 0.05% phosphoric acid in 80% acetonitrile, the flow rate was 1.25 mL/min, the sample volume was 20 μL, and the temperature was 35°C. The gradient was as follows: 10–25% B for 0–10 min, 25–40% B for 10–20 min, 40–80% B for 20–25 min, 80% B for 25–30 min, 80–10% B for 30–32 min, and 10% B for 32–40 min. The identification and quantification were performed at 360 nm with ellagic acid as the standard.

The concentrations of the ET metabolites were determined in the cecal digesta, urine and serum. A frozen sample of the digesta (0.5–1 g) was mixed with acetone (2 mL), sonicated for 10 min, and centrifuged (5 min, 10,000 *g*). Next, the supernatant was collected in a test tube. The procedure was repeated twice with 2 mL and 1 mL of 70% acetone. After the collection of the supernatant, the extract was concentrated using a vacuum concentrator (ScanSpeed 40, Labogene, Denmark) and then dissolved in methanol (1 mL). The ET metabolites were then determined using HPLC (a Knauer Smartline system with a photodiode array detector, Berlin, Germany) coupled with a Gemini C18 column (110 Å, 250×4.60 mm; 5 μm, Phenomenex, Torrance, USA). The separation conditions were the same as those used in the determination of the ETs in the dietary extracts. The ET metabolites were identified by comparisons of the UV spectra with the available data in the literature [4] and additionally confirmed with the MS method described below. A serum sample (0.5 mL) was mixed with acetone (1 mL), sonicated for 10 min, and centrifuged (5 min, 10,000 *g*). Next, the supernatant was collected in a test tube. The procedure was repeated, and both supernatants were collected in a test tube and concentrated using a vacuum concentrator (ScanSpeed 40, Labogene, Denmark). Next, the concentrated sample was dissolved in methanol (200 μL) and analyzed with HPLC-ESI-MS using a Dionex UltiMate 3000 UHPLC and a Thermo Scientific Q Exactive series quadrupole ion trap mass spectrometer. The ET metabolites were separated using a Kinetex C18 column (110 Å, 150×2.1 mm; 2.6 μm, Phenomenex, Torrance, USA) and a binary gradient of 0.1% formic acid in water (phase A) and 0.1% formic acid in acetonitrile (phase B) at a flow rate of 0.5 mL/min as follows: stabilization for 1.44 min with 5% B, 5–15% B for 1.44–2.98 min, 15–40% B for 2.98–10.1 min, 40–73% B for 10.1–11.5 min, 73% B for 11.55–12.7 min, 73–5% B for 12.7–13.28 min, and 5% B for 13.28–18 min. The MS analysis was performed in negative ion mode under the following conditions: capillary voltage, +4 kV; sheath gas pressure, 75 arbitrary units; auxiliary gas, 17 arbitrary units; and scan range, 120–1200 m/z. Urolithin-A isolated from human urine via semipreparative HPLC was used as the standard for the quantification of the ET metabolites. The detailed procedure of the urolithin-A isolation is described elsewhere [4]. A urine sample (2 mL) was added to the Strata-X 33μ column (Polymeric reversed phase 60 mg/3 mL, Phenomenex, Torrance, USA), rinsed with 1 M phosphoric acid buffer, eluted with 99.9% methanol (Sigma-Aldrich, St. Louis, MO, USA), and subsequently analyzed by HPLC-ESI-MS in the same manner as serum.

### Animal study

This study was carried out in strict accordance with the recommendations of the National Ethic Commission (Warsaw, Poland). All procedures and experiments complied with the guidelines and were approved by the Local Ethic Commission of the University of Warmia and Mazury (Olsztyn, Poland, Permit Number: 32/2012) with respect to animal experimentation and care of animals under study, and all efforts were made to minimize suffering. The nutritional experiment was performed on 48 male Wistar rats (aged 8 weeks) that were allocated into 6 groups of 8 animals each, and the rats were housed individually in metabolic plastic cages. The initial body weights were comparable between groups and averaged 257 ± 0.89 g. For 4 weeks, each group was fed with a modified version of the semi-purified rodent diet recommended by Reeves (1997) [18]. Animals were fed fresh diet everyday *ad libitum* with continuous access to distilled water. All experimental diets were similar in terms of dietary ingredients with the exceptions of the phenolic fraction and fiber content. Different dietary carbohydrates and protein amount provided by the EP and EPA extracts were negligible in comparison to the entire level of the carbohydrates and protein in the diets when the changes in the functioning of the gastrointestinal tract are considered. The fiber contents were from different sources, i.e., insoluble α-cellulose (SIGMA, Poznań, Poland) or a prebiotic FOS that was fermentable in the lower gut (Orafti, Oreye, Belgium). The CEL content in the standard-fiber diets was 6% (group C). In the prebiotic-supplemented diets, half of the dietary CEL was replaced with FOS. The strawberry extracts EP and EPA were added to diets with CEL and FOS in amounts of 0.28% and 0.70%, respectively (groups EP_CEL/FOS_ and EPA_CEL/FOS_). The total strawberry polyphenols in all of the experimental diets were similar. In all experimental diets, the strawberry phenolic extracts were added at the expense of cornstarch. Details regarding the proportional composition of each group-specific diet are displayed in Table 2. To represent a realistic amount of fresh strawberries consumed by humans, the diets used in this study were calculated with the aid of the body surface area normalization method [19] and literature data for polyphenol content in the strawberry. The individual body weights of the rats and their food intakes were recorded on weekly and daily bases, respectively. The animals were maintained under standard conditions at a temperature of 21–22°C and a relative air humidity of 50–70% with intensive room ventilation (15×/h) and a 12-h lighting regimen.

**Table 2:** Diet composition.

	C_CEL_	C_FOS_	EP_CEL_	EP_FOS_	EPA_CEL_	EPA_FOS_
	%					
Casein	14.8	14.8	14.8	14.8	14.8	14.8
Cellulose^1^	6	3	6	3	6	3
Fructooligosaccharides	0	3	0	3	0	3
Rapeseed oil	8	8	8	8	8	8
Mineral mix^2^	1	1	1	1	1	1
Vitamin mix^2^	3.5	3.5	3.5	3.5	3.5	3.5
Choline chloride	0.2	0.2	0.2	0.2	0.2	0.2
DL-methionine	0.2	0.2	0.2	0.2	0.2	0.2
Cholesterol	0.5	0.5	0.5	0.5	0.5	0.5
Extract EP	0	0	0.28	0.28	0	0
Extract EPA	0	0	0	0	0.70	0.70
Corn starch	65.8	65.8	65.52	65.52	65.10	65.10
Polyphenols*	0	0	0.199	0.199	0.198	0.198
Ellagitannins (ETs)*	0	0	0.121	0.121	0.096	0.096
Proanthocyanidins (PACs)*	0	0	0.069	0.069	0.071	0.071
Flavonols*	0	0	0.009	0.009	0.013	0.013
Anthocyanins (ACs)*	0	0	0	0	0.018	0.018
Metabolized carbohydrates*	0	0	0.05	0.05	0.46	0.46

*from extract EP or EPA.

^1^The α-cellulose preparation was obtained from Sigma-Aldrich (No. C8002).

^2^Recommended for the AIN-93G diet (Reeves, 1997) [18].

### Sample collection and analysis

At the termination of the experiment, the rats were anesthetized with sodium pentobarbital according to the recommendations for the euthanasia of laboratory animals (50 mg/kg body weight). After laparotomy, blood samples were collected from the *vena cava* and stored in tubes containing ethylenediaminetetraacetic acid. The small intestine, cecum and colon were removed and weighed. Immediately after euthanasia (ca. 10 min), the small intestine, cecal and colonic pH values were measured directly in the intestine segments (model 301 pH meter; Hanna Instruments, Vila do Conde, Portugal). The small intestine, cecum and colon were removed and weighed. Fresh cecal digesta were used for the determination of the ammonia content, which was extracted, trapped in a solution of boric acid, and quantified via direct titration with sulfuric acid [20]. The concentrations of SCFAs in the cecal digesta samples were determined by gas chromatography (Shimadzu GC-2010, Kyoto, Japan). The samples (0.2 g) were mixed with 0.2 mL of formic acid, diluted with deionized water and centrifuged at 7211 *g* for 10 min. The supernatant was loaded onto a capillary column (SGE BP21, 30 m × 0.53 mm) using an on-column injector. The initial oven temperature was 85°C and was increased to 180°C at 8°C/min and held at 180°C for 3 min. The temperatures of the flame ionization detector and the injector were 180°C and 85°C, respectively. The sample volume for GC analysis was 1 μL. The concentrations of cecal putrefactive SCFAs (PSCFAs) were calculated as the sum of isobutyric acid, isovaleric acid and valeric acid contents. All SCFAs analyses were performed in duplicate. Pure acetic, propionic, butyric, isobutyric, isovaleric and valeric acids were obtained from Sigma Co. (Poznan, Poland), and a mixture of these acids was used to create a standard plot from which the amounts of single acids were subsequently calculated. This additional set of pure acids was included in each GC run of samples at five sample intervals to maintain calibration. The activities of selected bacterial enzymes (β-glucosidase, β-galactosidase and β-glucuronidase) released into the cecal environment and in the feces were measured according to the rates of p- and o-nitrophenol release from their nitrophenyl glucosides according to a previously described method [21].

The blood was centrifuged for 15 min at 380 g and 4°C, and the obtained plasma was then stored at -70°C until analysis. The triglycerides (TG), and total cholesterol (TC), the fractions of HDL cholesterol (HDL) and LDL cholesterol (LDL), were estimated using a biochemical analyzer (Horiba, Pentra C200).

### Statistical analysis

The data are expressed as the means and pooled standard errors of the means (SEMs). The STATISTICA software version 10.0 (StatSoft Corp., Krakow, Poland) was used to determine whether the variables differed between the treatment groups. Two-way ANOVA was applied to assess the effects of the dietary addition of the strawberry extracts (i.e., no addition, extract EP and extract EPA; E), the type of diet in terms of the included fiber (i.e., the standard diet with 6% CEL and the diet with 3% FOS added in place of the 3% CEL; F), and the interaction between the investigated factors (E × F). When the ANOVA indicated significant treatment effects, the means were evaluated using Duncan’s multiple range tests. The data were checked for normality prior to the statistical analyses. Differences P<0.05 were considered significant. The results are presented as the mean values ± standard error of the mean (SEM).

## Results

Neither the addition of the strawberry extracts nor the dietary fiber type affected (P>0.05) the final gain (BWG) or dietary intake of rats during the 4-wk study (Table 3). A two-way ANOVA revealed that the dietary FOS significantly increased the relative full (i.e., with the intestinal contents) mass of the small intestine and caused a significant decrease in the pH of digesta in this segment of the gastrointestinal tract (in both cases P<0.001 vs CEL). The type of dietary fiber significantly affected the relative mass of the cecal tissue and the accumulated digesta (in both cases FOS>CEL, P<0.001). Interestingly, these effects of fiber type were not observed in the colon. The FOS treatment lowered the cecal ammonia concentration (P = 0.030 vs CEL). A two-way ANOVA revealed that the strawberry extract EPA led to an increase in the bulk of the cecal digesta compared with the control and EP dietary treatments (P = 0.024). The extract by fiber type interaction was significant for the dry mass (DM) concentration and the digesta pH in the cecum (P = 0.007 and P = 0.029, respectively). Compared with the control, the application of extract EP, but not EPA, significantly increased the cecal DM concentration when the diets with FOS were considered (P<0.05). In the CEL groups, this effect was noted in the EPA but not the EP extract. The dietary application of both strawberry extracts to the FOS but not the CEL diets significantly lowered the cecal pH value (P<0.05). FOS added to a diet effectively reduced the pH values of the digesta in both the rat cecum and colon (P<0.05).

**Table 3:** Body weight gain (BWG), diet intake and gastrointestinal tract indices of the rats fed the experimental diets*.

	BWG	Intake	Small intestine		Cecum					Colon		
			mass	pH	tissue	digesta	DM	NH_3_	pH	tissue	digesta	pH
Group (n = 8)	g	g	g/100 g BW		g/100 g BW	g/100 g BW	%	mg/g		g/100 g BW	g/100 g BW	
C_CEL_	91.0±2.52	503±7.0	1.87±0.044	7.46±0.119	0.173±0.005	0.501±0.031	23.8±0.77^b^^c^	0.257±0.010	7.59±0.087^a^	0.280±0.005	0.300±0.019	7.63±0.110
C_FOS_	85.8±3.64	489±8.5	1.97±0.042	6.68±0.092	0.220±0.005	0.858±0.024	23.4±0.50^c^	0.209±0.008	6.86±0.053^b^	0.290±0.012	0.271±0.025	7.11±0.064
EP_CEL_	84.2±4.75	502±7.4	1.85±0.041	7.09±0.130	0.165±0.002	0.524±0.042	24.4±0.62^b^^c^	0.237±0.023	7.42±0.064^a^	0.298±0.008	0.290±0.025	7.51±0.074
EP_FOS_	98.0±4.20	509±7.6	2.09±0.045	6.68±0.116	0.211±0.006	0.825±0.042	25.6±0.52^a^^b^	0.217±0.014	6.40±0.116^c^	0.306±0.014	0.307±0.036	6.87±0.143
EPA_CEL_	93.5±3.60	508±8.8	1.89±0.024	7.22±0.161	0.164±0.005	0.609±0.021	26.4±0.27^a^	0.227±0.010	7.55±0.096^a^	0.251±0.010	0.259±0.020	7.71±0.055
EPA_FOS_	82.1±6.80	493±10.2	2.08±0.038	6.71±0.102	0.233±0.009	0.946±0.057	23.5±0.63^c^	0.208±0.019	6.31±0.078^c^	0.272±0.009	0.275±0.025	6.87±0.126
Extract (E)												
C (without)	88.4	496	1.92	7.07	0.197	0.679^b^	23.6^b^	0.233	7.23^a^	0.285^a^	0.285	7.37
EP	91.1	506	1.97	6.88	0.188	0.674^b^	25.0^a^	0.227	6.91^b^	0.302^a^	0.299	7.19
EPA	87.8	501	1.98	6.96	0.198	0.778^a^	25.0^a^	0.218	6.93^b^	0.262^b^	0.267	7.29
*P value*	*0*.*762*	*0*.*558*	*0*.*281*	*0*.*350*	*0*.*192*	*0*.*024*	*0*.*042*	*0*.*622*	*0*.*001*	*0*.*002*	*0*.*519*	*0*.*254*
Fiber (F)												
CEL	89.6	504	1.87^b^	7.26^a^	0.167^b^	0.545^b^	24.9	0.241^a^	7.52^a^	0.276	0.283	7.62^a^
FOS	88.6	497	2.05^a^	6.69^b^	0.221^a^	0.876^a^	24.2	0.211^b^	6.53^b^	0.289	0.284	6.95^b^
*P value*	*0*.*798*	*0*.*317*	*<0*.*001*	*<0*.*001*	*<0*.*001*	*<0*.*001*	*0*.*165*	*0*.*030*	*<0*.*001*	*0*.*140*	*0*.*951*	*<0*.*001*
Interaction E×F												
*P value*	*0*.*071*	*0*.*379*	*0*.*228*	*0*.*355*	*0*.*109*	*0*.*786*	*0*.*007*	*0*.*588*	*0*.*029*	*0*.*812*	*0*.*641*	*0*.*318*

Values are expressed as mean ± standard error of mean.

*C_CEL_, control diet with 6% cellulose (CEL) as the dietary fiber; C_FOS_, control diet with 3% fructooligosaccharides (FOS) and 3% cellulose as the dietary fiber; EP_CEL_, diet with the EP strawberry extract and CEL as the dietary fiber; EP_FOS_, diet with the EP extract and FOS/CEL as the dietary fiber; EPA_CEL_, diet with the EPA strawberry extract and CEL as the dietary fiber; EPA_FOS_, diet the EPA extract and FOS/CEL as the dietary fiber.

^a,b,c^ The mean values within a column with different superscript letters were significantly different (P<0.05). The differences between the C_CEL_, C_FOS_, EP_CEL_, EP_FOS_, EPA_CEL_, and EPA_FOS_ groups are indicated with superscripts only in the cases of statistically significant E×F interactions (P<0.05).

**Fig 1:**
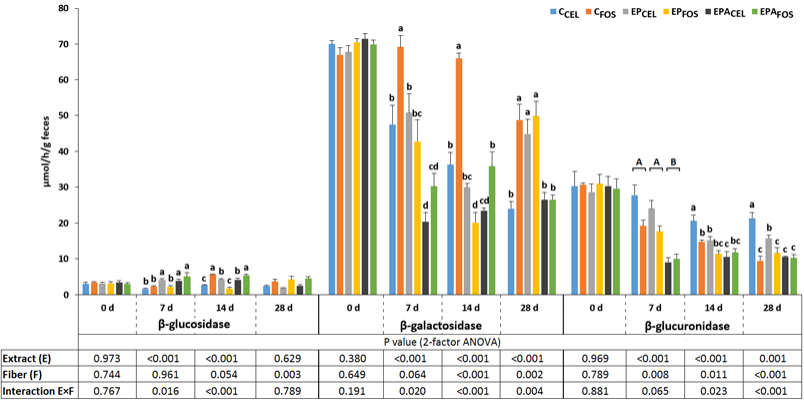
Fecal extracellular bacterial enzyme activity during the experimental feeding of the rats. C_CEL_, control diet with 6% cellulose (CEL) as the dietary fiber; C_FOS_, control diet with 3% fructooligosaccharides (FOS) and 3% cellulose as the dietary fiber; EP_CEL_, diet with the EP strawberry extract and CEL as the dietary fiber; EP_FOS_, diet with the EP extract and FOS/CEL as the dietary fiber; EPA_CEL_, diet with the EPA strawberry extract and CEL as the dietary fiber; EPA_FOS_, diet the EPA extract and FOS/CEL as the dietary fiber. Values are expressed as mean ± standard error of mean. ^A,B^ The significant differences between the extracts and control groups (P<0.05). ^a,b^ The significant differences between the C_CEL_, C_FOS_, EP_CEL_, EP_FOS_, EPA_CEL_, and EPA_FOS_ groups are indicated with superscripts only cases of statistically significant E×F interactions (P<0.05).

The differences in the types of strawberry extracts and fibers added to the diet caused changes in the extracellular activities of some bacterial enzymes in the feces of the rats during the study (Fig 1). After the first week of the experiment, the fecal β-glucosidase activity was significantly increased by both extracts with the exception of the EP extract in the rats that were fed FOS-containing diet (i.e., the E×F interaction was significant, P = 0.016). An extract by fiber type interaction was also observed after the second week of experimental feeding. Both extracts caused a significant increase in the β-glucosidase activity in the CEL but not the FOS groups (P<0.05). Regarding the FOS dietary environment, only the EP extract decreased the activity of this enzyme (P<0.05). The type of fiber affected the fecal β-glucosidase activity after the fourth week of the experiment (FOS>CEL, P = 0.003). Regarding the fecal bacterial β-galactosidase, extract by fiber type interactions were noted on days 7, 14, and 28 of the study. After the first and second week, the dietary applications of both strawberry extracts significantly reduced the activity of bacterial β-galactosidase in the FOS groups, and in the dietary CEL conditions (P<0.05), this effect was observed for the EPA extract but not the EP extract. After the fourth week, the EPA extract reduced the activity of this enzyme on the dietary FOS (P<0.05 vs C_FOS_ and EP_FOS_), whereas the EP extract significantly increased the β-galactosidase activity in the CEL treatment (P<0.05 vs C_FOS_ and EPA_FOS_). The extract application by fiber type interactions (on days 14 and 28) revealed that in the dietary CEL environment, both extracts significantly decreased the β-glucuronidase activity (P<0.05), and the EPA extract exerted a greater effect than the EP extract. Irrespective of the strawberry extract contents in the diet, the addition of FOS to the diet effectively reduced the activity of bacterial β-glucuronidase in the feces of the rats during the entire experimental period (P<0.01).

**Fig 2:**
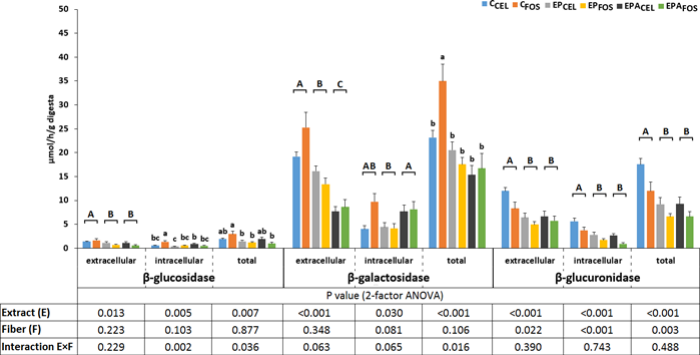
Cecal bacterial enzyme activities and enzyme release rates into the intestinal environments of the rats. C_CEL_, control diet with 6% cellulose (CEL) as the dietary fiber; C_FOS_, control diet with 3% fructooligosaccharides (FOS) and 3% cellulose as the dietary fiber; EP_CEL_, diet with the EP strawberry extract and CEL as the dietary fiber; EP_FOS_, diet with the EP extract and FOS/CEL as the dietary fiber; EPA_CEL_, diet with the EPA strawberry extract and CEL as the dietary fiber; EPA_FOS_, diet the EPA extract and FOS/CEL as the dietary fiber. Values are expressed as mean ± standard error of mean. ^A,B^ The significant differences between the extracts and control groups (P<0.05). ^a,b^ The significant differences between the C_CEL_, C_FOS_, EP_CEL_, EP_FOS_, EPA_CEL_, and EPA_FOS_ groups are indicated with superscripts only cases of statistically significant E×F interactions (P<0.05).

The activities of the bacterial enzymes β-glucosidase, β-galactosidase, and β-glucuronidase that were released into the cecal environment were significantly reduced by the dietary application of both strawberry extracts (P<0.05) (Fig 2). Regarding extracellular β-galactosidase, extract EPA was more effective than extract EP (P<0.001). The extract by fiber type interactions were significant in terms of the intracellular and total β-glucosidase cecal activities (P = 0.002 and P = 0.036, respectively). The nature of these interactions involved a decrease in the activity of β-glucosidase in the FOS and not in the CEL groups. Irrespective of the fiber type, a two-way ANOVA revealed that the highest and lowest extracellular bacterial β-galactosidase activities were noted in the control and EPA treatments, respectively (both cases, P<0.05 vs the other treatments). The total β-galactosidase activity was highest in the C_FOS_ rats (P<0.05 vs all other groups, irrespective of fiber type; i.e., the E×F interaction was significant). The extracellular and intracellular activities of bacterial β-glucuronidase were efficiently reduced by the addition of both strawberry extracts as well as FOS to the diet (P<0.001).

The concentrations of SCFAs differed significantly according to the applied dietary treatment (Table 4). Compared with the CEL fiber type, dietary FOS significantly increased the total SCFA concentration in the cecum (P = 0.001), primarily due to the increase in acetic acid level elicited by FOS and despite the opposite effect of dietary FOS on the putrefactive SCFAs (P<0.001). Similar extract by fiber interactions were observed in the propionic and butyric acid concentrations in the cecal digesta. The applications of both extracts failed to alter the C3 or C4 concentrations in the CEL diet treatments but significantly reduced these levels in the FOS treatments (P<0.01). Regarding propionate, the decrease observed in the EPA_FOS_ group significantly differed from that in the EP_FOS_ group (P<0.05). Regardless of fiber type, the application of the EPA extract but not the EP extract significantly reduced the total cecal SCFA concentration (P<0.05). Both extracts reduced the cecal concentrations of putrefactive SCFAs, but the EPA extract elicited a greater reduction than the EP extract (P<0.001). The profile analysis of the three major fatty acids, i.e., acetic acid, propionic acid, and butyric acid, revealed that both groups treated with strawberry extracts and the groups with FOS were characterized by significantly greater acetic acid:total SCFA ratios than the controls (P<0.001). The opposite effect was noted for butyric acid in the EPA treatment (P<0.01). The extract by fiber type interaction revealed that both extracts significantly reduced the propionic acid:total SCFA ratio in the dietary FOS environment (P<0.001) but not in the dietary CEL environment.

**Table 4:** Short chain fatty acid (SCFA) concentrations, total pools and profiles in the cecum digesta during the experimental feeding of the rats*.

	SCFA concentration								SCFA profile		
	C2^1^	C3	C4i	C4	C5i	C5	PSCFA	SCFA	C2	C3	C4
Group (n = 8)	μmol/g digesta								% of total SCFA		
C_CEL_	65.1±1.67	16.9±0.70^a^^b^	1.77±0.107	9.49±0.84^b^	2.05±0.147	2.06±0.152	5.88±0.277	97.4±2.78	66.9±0.96	17.4±0.36^a^	9.66±0.730
C_FOS_	88.7±3.65	19.3±0.94^a^	1.55±0.269	13.9±1.49^a^	1.18±0.075	1.46±0.126	4.19±0.354	126±4.70	70.4±1.35	15.3±0.42^a^	11.0±1.117
EP_CEL_	78.9±5.17	17.0±0.59^a^^b^	2.55±0.665	10.3±0.95^b^	1.45±0.298	1.43±0.166	5.43±0.733	112±6.20	70.3±0.99	15.4±0.42^a^	9.35±0.831
EP_FOS_	94.3±7.86	15.1±1.15^b^	0.97±0.154	10.4±1.12^b^	0.78±0.101	0.83±0.067	2.58±0.217	122±8.96	76.7±1.27	12.5±0.74^b^	8.60±0.795
EPA_CEL_	64.1±3.26	16.0±1.36^a^^b^	1.06±0.125	8.40±0.95^b^	1.07±0.093	1.47±0.112	3.59±0.313	92.1±5.22	69.8±1.27	17.4±1.11^a^	8.97±0.517
EPA_FOS_	83.1±4.65	11.6±1.05^c^	0.61±0.131	7.03±0.98^b^	1.10±0.343	0.65±0.120	2.36±0.357	104±5.02	79.8±1.99	11.2±0.92^b^	6.73±0.856
Extract (E)											
C (without)	76.9^a^^b^	18.1^a^	1.66^a^	11.7^a^	1.61^a^	1.76^a^	5.03^a^	112^a^	68.7^b^	16.3^a^	10.3^a^
EP	86.6^a^	16.1^a^	1.76^a^	10.4^a^	1.11^b^	1.13^b^	4.01^b^	117^a^	73.5^a^	14.0^b^	8.98^a^^b^
EPA	73.6^b^	13.8^b^	0.83^b^	7.72^b^	1.08^b^	1.06^b^	2.98^c^	98.1^b^	74.8^a^	14.3^b^	7.85^b^
*P value*	*0*.*040*	*0*.*001*	*0*.*015*	*0*.*004*	*0*.*033*	*<0*.*001*	*<0*.*001*	*0*.*011*	*<0*.*001*	*0*.*007*	*0*.*028*
Fiber (F)											
CEL	69.3^b^	16.7	1.79^a^	9.40	1.52^a^	1.65^a^	4.96^a^	100^b^	69.0^b^	16.7^a^	9.33
FOS	88.7^a^	15.3	1.04^b^	10.5	1.02^b^	0.98^b^	3.04^b^	118^a^	75.6^a^	13.0^b^	8.76
*P value*	*<0*.*001*	*0*.*131*	*0*.*009*	*0*.*264*	*0*.*007*	*<0*.*001*	*<0*.*001*	*0*.*001*	*<0*.*001*	*<0*.*001*	*0*.*441*
Interaction E×F											
*P value*	*0*.*725*	*0*.*010*	*0*.*105*	*0*.*041*	*0*.*109*	*0*.*635*	*0*.*180*	*0*.*280*	*0*.*083*	*0*.*026*	*0*.*143*

Values are expressed as mean ± standard error of mean.

*C_CEL_, control diet with 6% cellulose (CEL) as the dietary fiber; C_FOS_, control diet with 3% fructooligosaccharides (FOS) and 3% cellulose as the dietary fiber; EP_CEL_, diet with the EP strawberry extract and CEL as the dietary fiber; EP_FOS_, diet with the EP extract and FOS/CEL as the dietary fiber; EPA_CEL_, diet with the EPA strawberry extract and CEL as the dietary fiber; EPA_FOS_, diet the EPA extract and FOS/CEL as the dietary fiber.

^a,b,c^ The mean values within a column with different superscript letters were significantly different (P<0.05). The differences between the C_CEL_, C_FOS_, EP_CEL_, EP_FOS_, EPA_CEL_, and EPA_FOS_ groups are indicated with superscripts only in cases of statistically significant E×F interactions (P<0.05).

PSCFA, putrefactive SCFA (the sum of the isobutyric, isovaleric and valeric acids)

^1^C2, acetate; C3, propionate; C4i, isobutyrate; C4, butyrate; C5i, isovalerate; C5, valerate.

The additions of either dietary strawberry extract and the addition of FOS failed to affect (P>0.05) the blood serum total cholesterol or the HDL and LDL fractions, (Table 5).

**Table 5:** Lipid profiles (mmol/L) of the blood sera of the rats fed the experimental diets*.

Group (n = 8)	TC	HDL	LDL	TG
C_CEL_	2.23±0.024	0.598±0.017	0.476±0.025	1.50±0.088
C_FOS_	2.25±0.122	0.636±0.014	0.397±0.034	1.80±0.070
EP_CEL_	2.24±0.111	0.604±0.024	0.480±0.033	1.40±0.106
EP_FOS_	2.44±0.121	0.673±0.050	0.511±0.037	1.69±0.087
EPA_CEL_	2.40±0.066	0.619±0.038	0.490±0.034	1.53±0.162
EPA_FOS_	2.26±0.125	0.626±0.056	0.480±0.026	1.69±0.133
Extract (E)				
C (without)	2.24	0.617	0.436	1.65
EP	2.34	0.638	0.496	1.55
EPA	2.33	0.623	0.485	1.61
*P value*	*0*.*610*	*0*.*854*	*0*.*189*	*0*.*667*
Fiber (F)				
CEL	2.29	0.607	0.482	1.48^b^
FOS	2.31	0.645	0.463	1.73^a^
*P value*	*0*.*766*	*0*.*244*	*0*.*495*	*0*.*015*
Interaction E×F				
*P value*	*0*.*316*	*0*.*743*	*0*.*274*	*0*.*798*

Values are expressed as mean ± standard error of mean.

*C_CEL_, control diet with 6% cellulose (CEL) as the dietary fiber; C_FOS_, control diet with 3% fructooligosaccharides (FOS) and 3% cellulose as the dietary fiber; EP_CEL_, diet with the EP strawberry extract and CEL as the dietary fiber; EP_FOS_, diet with the EP extract and FOS/CEL as the dietary fiber; EPA_CEL_, diet with the EPA strawberry extract and CEL as the dietary fiber; EPA_FOS_, diet the EPA extract and FOS/CEL as the dietary fiber.

^a,b^ The mean values within a column with different superscript letters were significantly different (P<0.05).

TG, triacylglycerols; TC, total cholesterol; HDL, HDL-cholesterol; LDL, LDL-cholesterol.

As shown in Table 6, ellagitannin metabolites appeared in the cecal digesta, urine and serum following the dietary application of the EP and EPA strawberry extracts. Two-way ANOVA analyses revealed that the cecal and urinal metabolite levels increased following the consumption of the diets with EPA compared to the consumption of those with EP (P<0.05). Moreover, the extract by fiber type interactions were significant for the urolithin A and nasutin A concentrations in the cecum and for the urolithin A glucuronide and nasutin A levels in the urine (P<0.05). These interactions were driven by the appearance of significantly greater metabolite concentrations following the FOS treatments than the CEL treatments when the EPA extract was considered (P<0.05). These differences were not observed for the EP extract.

**Table 6:** Ellagitannin metabolite profiles in the cecal digesta, urine and serum of the rats fed the experimental diets*.

	Cecal digesta			Urine			Serum	
	Urolithin A	Nasutin A	Released EA	Urolithin A glucuronide	Nasutin A glucuronide	Nasutin A	Urolithin A	Isonasutin A glucuronide
Group (n = 8)	μg/g			μg/mL			μg/mL	
C_CEL_	0.00±0^c^	0.00±0^c^	0.00±0	0.000±0^b^	0.000±0	0.000±0^c^	0.00±0	0.00±0
C_FOS_	0.00±0^c^	0.00±0^c^	0.00±0	0.000±0^b^	0.000±0	0.000±0^c^	0.00±0	0.00±0
EP_CEL_	0.710±0.436^b^^c^	9.08±2.090^b^^c^	1541±133	0.012±0.007^b^	0.073±0.027	0.072±0.006^b^	0.158±0.022	0.013±0.001
EP_FOS_	1.34±0.405^a^^b^	26.0±4.329^b^	1517±116	0.060±0.028^b^	0.106±0.050	0.058±0.005^b^	0.208±0.018	0.012±0.001
EPA_CEL_	0.182±0.121^b^^c^	21.4±4.584^b^	1767±53.6	0.000±0^b^	0.136±0.060	0.071±0.011^b^	0.174±0.032	0.011±0.001
EPA_FOS_	2.23±0.678^a^	62.6±14.58^a^	1848±95.6	0.543±0.250^a^	0.673±0.401	0.116±0.019^a^	0.204±0.028	0.011±0.001
Extract (E)								
C (without)	0.00^b^	0.00^c^	0.00^c^	0.000^b^	0.000	0.000^c^	0.000^b^	0.000^b^
EP	1.02^a^	17.5^b^	1529^b^	0.036^b^	0.089	0.065^b^	0.183^a^	0.012^a^
EPA	1.20^a^	42.0^a^	1808^a^	0.272^a^	0.405	0.094^a^	0.188^a^	0.011^a^
*P value*	*0*.*008*	*<0*.*001*	*<0*.*001*	*0*.*046*	*0*.*083*	*<0*.*001*	*<0*.*001*	*<0*.*001*
Fiber (F)								
CEL	0.298^b^	10.1^b^	1103	0.004^b^	0.070	0.048	0.111	0.008
FOS	1.19^a^	29.5^a^	1122	0.201^a^	0.260	0.058	0.137	0.007
*P value*	*0*.*009*	*0*.*002*	*0*.*800*	*0*.*040*	*0*.*212*	*0*.*242*	*0*.*153*	*0*.*702*
Interaction E×F								
*P value*	*0*.*040*	*0*.*019*	*0*.*833*	*0*.*041*	*0*.*273*	*0*.*021*	*0*.*540*	*0*.*867*

Values are expressed as mean ± standard error of mean.

*C_CEL_, control diet with 6% cellulose (CEL) as the dietary fiber; C_FOS_, control diet with 3% fructooligosaccharides (FOS) and 3% cellulose as the dietary fiber; EP_CEL_, diet with the EP strawberry extract and CEL as the dietary fiber; EP_FOS_, diet with the EP extract and FOS/CEL as the dietary fiber; EPA_CEL_, diet with the EPA strawberry extract and CEL as the dietary fiber; EPA_FOS_, diet the EPA extract and FOS/CEL as the dietary fiber.

^a,b,c^ The mean values within a column with different superscript letters were significantly different (P<0.05). The differences between the C_CEL_, C_FOS_, EP_CEL_, EP_FOS_, EPA_CEL_, and EPA_FOS_ groups are indicated with superscripts only in cases of statistically significant E×F interactions (P<0.05).

## Discussion

The results of the present study indicated that the EP strawberry extract obtained from strawberry press cakes was a valuable source of the polyphenols and consisted of 71.8 g/100 g phenolic compounds, most of which were ETs and PACs. The second EPA strawberry extract was made from juice, contained a lower total phenolic content (28.6 g/100 g) and was a source of ETs, PACs and ACs. Other studies of polyphenolic strawberry extracts have reported a very wide range of the total phenolic content from 28.8 [22] to 59.3 g/100 g [4], and the reported key polyphenol contents, e.g., ETs, PACs and ACs, are also different. The concentrations of these bioactive substances are strongly influenced by genotypic and extrinsic factors, such as agricultural practices, the environment and maturity [23].

The addition of FOS to the diets significantly increased the production of the bacterial SCFAs; thus, the digesta from the small intestine, cecum and colon were more acidic. Lower digesta pH values promote positive microbiota proliferation and decrease the growth of pathogenic bacterial species [24]. The susceptibility of CEL to the fermentative processes that occur in the hindgut is markedly lower than that of FOS, which has been proven to possess prebiotic properties and to enhance the production of SCFAs [25]. The greater fermentation process activities might explain the considerably greater masses of the cecal tissues and digesta in the rats that were fed the diets with FOS. The consumption of the diets with FOS beneficially increased ammonia utilization. Decreased ammonia concentrations are considered a positive change because this compound can destroy cells, alter nucleic acid synthesis and increase virus infections in the lower bowel [26]. In contrast, the beneficial FOS-induced fermentation process was partially reduced by the EPA extract. This influence was visible in significantly lower concentrations of some SCFA e.g., butyrate acid and significantly reduced mass of the colon tissue. Butyrate is an energy substrate for colonocytes [1]; therefore, the significantly lower concentrations of this SCFA observed in in the EPA extract group might explain the reduced mass of the colon tissue. Furthermore, ETs were the main polyphenolic compounds of both of the strawberry extracts, and ETs release ellagic acid (EA) upon hydrolysis [27]; thus, the poor solubility of EA [28] may reduce the amount of bound water and increase the amount of the dry matter in the cecal digesta. Indeed, the amounts of released EA in the cecum in the groups that received the strawberry polyphenolic extracts were significantly higher than those in the control groups.

The effects of enhanced bacterial SCFA production following the administration of FOS were primarily associated with an increase in the acetic acid concentration. It is well-known that prebiotic FOS stimulate growth of beneficial bacteria in the lower gut, e.g. *Bifidobacteria*, that in turn are producers of acetic acid in large amounts, that is, larger than the amounts secreted by other probiotic species [1]. Some other probiotic bacteria that live in the gastrointestinal tract, e.g., *Lactobacillus plantarum* species, are known to ferment pentoses and/or gluconate to lactic and acetic acids [29]. Therefore, the addition of FOS interacted with the resident microbiota in a manner that enhanced acetic acid production capacity. Furthermore, acetate could be utilized by bacteria for the production of butyrate in the colon [30], which might explain the significantly higher concentration of butyric acid and lower concentration of acetic acid in control group with cellulose. However, the addition of the EPA extract, resulted in reductions in the concentrations of all of the SCFAs. The EPA extract contained ACs, which has antibacterial properties against some groups of bacteria that live in the gastrointestinal tract [10, 31], and thus might had effect on the SCFAs production. Nevertheless, both of the strawberry extracts significantly reduced the production of PSCFAs, which may be suggestive of less intensive anaerobic bacterial polypeptide and amino acid fermentation [21]. This beneficial effect might be related to the presence of the ETs and their derived metabolites, which possess antimicrobial and prebiotic effects [8]. A similar effect was observed in a study of Wistar rats that were fed diets containing high levels of ETs extracted from strawberries [4].

The modulation of microbial activity was also indicated by the changes in the bacterial enzyme activities in the cecal digesta and feces. Kosmala et al. (2014) [22] revealed that the inclusion of 5% FOS significantly decreases β-glucosidase and β-glucuronidase activities, whereas the activity of β-galactosidase is not significantly altered compared to a cellulose-enriched diet. In the present study, the addition of 3% FOS to the diets significantly decreased the activities of extracellular β-glucuronidase in the cecum and feces. This enzyme is characteristic of harmful bacteria species, and it has deconjugative properties that support the transformation of xenobiotics into more toxic substances [2, 32]. The extracellular activities of β-glucosidase and β-galactosidase were also considerable lower in in the cecum digesta following the addition of the strawberry polyphenolic extracts to the diets. Some studies have reported that ETs and PACs in the diet may modulate the activities of the microbiota and digestive enzymes [4, 11, 33, 34]. ETs and PACs in the diet can be assumed to selectively modulate the composition of the microbiota and thus decrease bacterial enzymatic activity. Furthermore, microbiota-derived metabolites (e.g., urolithin A) may exert antipathogenic effects in the colon and may thus contribute to maintaining the microbial equilibrium in the gut [35]. In the present study, the examination of polyphenolic extracts revealed that the groups that were fed FOS exhibited considerably greater higher concentrations of the urolithin A in the cecal digesta, which might the reduced activities of β-glucuronidase in the cecum digesta and feces. Furthermore, the extracts used in this study were also sources of flavonoids, which can decrease the activities of bacterial β-glucosidase and β-galactosidases in the cecal digesta of rats when applied as dietary supplements [36]. It was also observed differences in the activity of the bacterial enzymes between groups that received the EPA or EP extract. The bacterial catabolism of ACs involves the cleavage of 3-glycosidic linkages by bacterial β-glucosidase [10], and the presence of the ACs might have stimulated the bacteria to increase the activity of this enzyme. Indeed, after 7 days of the dietary experiment, considerably higher extracellular β-glucosidase activities were observed in the feces of the groups that received the EPA extract compared with those that received the EP extract. The EPA extract was also more effective in reducing the extracellular β-galactosidase and β-glucuronidase activities in the feces than the EP extract.

FOS are a well-known dietary ingredients that modulate activity of the microbiota [4]. Higher activity of the gastrointestinal microbiota elevate metabolism and absorption of the ETs derived from the diet and thus may exhibit systemic modulatory effects [3,4]. Some authors have found that urolithins, which are ET metabolites, are responsible for reductions in serum and liver lipids [6, 37]. Moreover, studies with animal models have demonstrated that supplementation with foods containing polyphenols can favorably reduce the plasma concentrations of total cholesterol, LDL-cholesterol, and TG and increase HDL-cholesterol concentrations plasma [11]. In the present study, the combination of FOS and strawberry polyphenolic extracts significantly increased metabolism of the ETs and thus concentrations of the urolithin A in the cecal digesta and the serum was considerable higher than in groups with CEL; however, differences in the serum lipid profile were not observed between the groups.

In summary, a previous study of rats demonstrated that ETs from strawberry extracts thwart the positive effects of FOS in the gastrointestinal tract [4]. In the present experiment, strawberry extract EPA containing ETs, PACs and also ACs in combination with FOS more effectively reduced β-glucuronidase activity in the feces and the production of propionic acid and PSCFAs in the cecum than the diets that included the EP extract without ACs. Moreover, compared to cellulose, the addition of dietary FOS increased the metabolism of the examined strawberry extracts in the cecum and therefore increased the concentrations of the metabolites in the cecal digesta and urine. Overall, the strawberry polyphenolic extracts modulated the effect of dietary FOS in the gastrointestinal tract; however, the combination of FOS and the ACs-containing EPA extract elicited greater beneficial effects in the lower gut environment than the EP extract.

## Supporting Information

S1 Table Undefined ellagitannin metabolites in the cecal digesta of the rats fed the experimental diets. (DOC)
