# Supplementary material for: Anthocyanins in Strawberry Polyphenolic Extract Enhance the Beneficial Effects of Diets with Fructooligosaccharides in the Rat Cecal Environment
Source: PLoS One. 2016 Feb 16;11(2):e0149081. doi: 10.1371/journal.pone.0149081 (PMC4755607; doi:10.1371/journal.pone.0149081)
Supplement: S1 Table — (DOC) [file pone.0149081.s001.doc]

# **Supporting Information**

Table 1. Undefined ellagitannin metabolites in the cecal digesta of the rats fed the experimental diets^*^.

|  | Metabolite 1 | Metabolite 2 | Metabolite 3 |
| --- | --- | --- | --- |
| Group (n=8) |  | µg/g |  |
| C_CEL_ | 0.00^d^ | 0.00 | 0.00^b^ |
| C_FOS_ | 0.00^d^ | 0.00 | 0.00^b^ |
| EP_CEL_ | 6.33^bc^ | 0.664 | 0.00^b^ |
| EP_FOS_ | 8.61^ab^ | 0.00 | 0.499^a^ |
| EPA_CEL_ | 5.32^c^ | 0.00 | 0.00^b^ |
| EPA_FOS_ | 10.9^a^ | 0.00 | 0.00^b^ |
| *SEM* | *0.685* | *0.077* | *0.048* |
| Extract (E) |  |  |  |
| C (without) | 0.00^b^ | 0.000 | 0.000^b^ |
| EP | 7.47^a^ | 0.332 | 0.249^a^ |
| EPA | 8.11^a^ | 0.000 | 0.000^b^ |
| *P value* | *<0.001* | *0.110* | *0.027* |
| Fiber (F) |  |  |  |
| CEL | 3.88^b^ | 0.221 | 0.000 |
| FOS | 6.50^a^ | 0.000 | 0.166 |
| *P value* | *<0.001* | *0.135* | *0.054* |
| Interaction E×F |  |  |  |
| *P value* | *0.011* | *0.110* | *0.027* |

*C_CEL_, control diet with 6% cellulose (CEL) as the dietary fiber; C_FOS_, control diet with 3% fructooligosaccharides (FOS) and 3% cellulose as the dietary fiber; EP_CEL_, diet with the EP strawberry extract and CEL as the dietary fiber; EP_FOS_, diet with the EP extract and FOS/CEL as the dietary fiber; EPA_CEL_, diet with the EPA strawberry extract and CEL as the dietary fiber; EPA_FOS_, diet the EPA extract and FOS/CEL as the dietary fiber.

^a,b^ The mean values within a column with different superscript letters are significantly different (P<0.05). The differences between the C_CEL_, C_FOS_, EP_CEL_, EP_FOS_, EPA_CEL_, and E. PA_FOS_ groups are indicated with superscripts only in cases of statistically significant E×F interactions (P<0.05).

Metabolite 1: UV 219, 268, 347, 466; RT=17.8 min. Metabolite 2: UV 214, 259, 290, 348; RT=25.7 min. Metabolite 3: UV 218, 232, 278, 303, 343; RT=26,5 min.

Table 2. UV spectra, [M – H]^-^, and HPLC retention times of the different ellagitannin metabolites in the urine and serum.

|  | Retention time, min | MS [M – H]^-^ | UV spectra |
| --- | --- | --- | --- |
| Urolithin A glucuronide | 6.74 | 403 | 216, 279, 295, 304, 349 |
| Nasutin A glucuronide | 6.99 | 445 | 222, 279, 315, 367, 379 |
| Isonasutin A glucuronide | 8.08 | 445 | 273, 310, 357, 367 |
| Urolithin A | 9.54 | 227 | 197, 218, 280, 307, 355 |
| Methylurolithin A | 9.98 | 243 [M – H]^+^ | 204, 278, 285, 333 |
| Nasutin A | 10.08 | 269 | 227, 245, 284, 323, 389 |
